# Coronavirus disease 2019 and cardiovascular complications: focused clinical review

**DOI:** 10.1097/HJH.0000000000002819

**Published:** 2021-02-26

**Authors:** Sahrai Saeed, Marijana Tadic, Terje H. Larsen, Guido Grassi, Giuseppe Mancia

**Affiliations:** a Department of Heart Disease, Haukeland University Hospital, Bergen, Norway; b Department of Cardiology, University Hospital ‘Dr Dragisa Misovic-Dedinje’, Belgrade, Serbia; c Department of Medicine and Surgery, University of Milano-Bicocca, Milan; d University of Milano-Bicocca, Milano and Policlinico di Monza, Monza, Italy

**Keywords:** acute cardiac injury, antihypertensive treatment, coronavirus disease 2019, hypertension, prognosis

## Abstract

The coronavirus disease 2019 (COVID-19) may cause not only an acute respiratory distress syndrome (ARDS) but also multiple organ damage and failure requiring intensive care and leading to death. Male sex, advanced age, chronic lung disease, chronic kidney disease and cardiovascular disease, such as hypertension, diabetes and obesity have been identified as risk factors for the COVID-19 severity. Presumably, as these three cardiovascular risk factors are associated with a high prevalence of multiorgan damage. In the present focused clinical review, we will discuss the cardiovascular complications of COVID-19 including acute cardiovascular syndrome (acute cardiac injury/COVID cardiomyopathy, thromboembolic complications and arrhythmias) and post-COVID-19 sequelae. Preliminary data shows that the cause of acute cardiovascular syndrome may be multifactorial and involve direct viral invasion of the heart and vascular system, as well as through the immune and inflammation-mediated systemic cytokine storm. COVID-19 survivors may also show persistently elevated blood pressure and sinus tachycardia at rest. Furthermore, poor diabetic control, persistent renal damage and cerebral sequelae, such as persistent cognitive and neuropsychiatric alterations are also frequently reported. A particular attention should be paid towards cardiovascular protection in COVID-19 patients who develop acute cardiovascular syndromes during hospitalization, and/or permanent/semipermanent sequelae after recovery from COVID-19. These conditions may require careful clinical assessment, treatment and close follow-up to avoid short-term and long-term complications.

## INTRODUCTION

The coronavirus disease 2019 (COVID-19) caused by the coronavirus 2 [severe acute respiratory syndrome coronavirus (SARS-CoV-2)], is a life-threatening disease, which may cause severe pneumonia and lung injury [acute respiratory distress syndrome (ARDS)] with subsequent multiple organ failure requiring intensive care and ventilation support [1–5]. The first official case of COVID-19 was discovered in the city of Wuhan, China, in December 2019. Since its outbreak, the virus rapidly spread to the entire world and in March 2020 the WHO officially declared a pandemic. At the time of writing, a total of 81 358 297 confirmed cases and 1 776 064 deaths have been registered worldwide [6]. COVID-19 had, and is still having, a devastating impact on virtually all aspects of human life, from the healthcare system to economy, social relations and mental health.

In the present focused clinical review, we will discuss the cardiovascular complications of COVID-19 during hospitalization, as well as the occurrence of post-COVID sequelae, with particular focus on major cardiovascular risk factors, such as hypertension and diabetes, and organ damages, such as those involving the heart and the kidneys.

## CORONAVIRUS DISEASE 2019, CARDIOVASCULAR RISK FACTORS AND ORGAN DAMAGE

After the outbreak of COVID-19, the initial reports suggested that male sex, older age and cardiovascular risk factors, such as hypertension, diabetes and obesity were common in COVID-19 patients [4,7,8]. This has been confirmed in most subsequent studies, which have also shown an association of the severity of COVID-19 not only with cardiovascular risk factors but also with the presence of risk factor-related organ damage. Roughly 40–50% of hospitalized COVID-19 patients have been shown to have underlying cardiovascular or cerebrovascular diseases [9,10], and an association has been found also between the severity of COVID-19 infection and acute cardiovascular damage [11]. A study from Northern Italy showed that among 99 hospitalized COVID-19 patients, 53 had a history of cardiac disease whereas only 46 were cardiac disease-free. Those with concomitant cardiac disease (40% with heart failure, 36% with atrial fibrillation and 30% with coronary artery disease) had a much poorer prognosis because of higher mortality, thromboembolic events and septic shock rates compared with patients without a history of cardiac disease [10]. Another study from northern Italy has shown that in more than 6200 COVID-19-infected people, history of hospitalization for cardiovascular events was 28% more common than in more than 31 000 control-matched for age, sex and municipality of residence [12]. Studies from the United States have reported similar results [5]. In the Yale COVID-19 Cardiovascular Registry, more than 40% of patients hospitalized with COVID-19 presented with a cardiovascular disease, such as coronary artery disease, heart failure and atrial fibrillation. As in previous studies, hypertension, type 2 diabetes and dyslipidemia were common cardiovascular risk factors [5], and in-hospital cardiovascular events were by no means rare, atrial fibrillation being detected in 19% of the patients, myocardial infarction in 17% and acute decompensated heart failure in 14%. Overall, 18% of the patients died in hospital and 39% experienced a major adverse cardiovascular event after admission. Nearly one-third of the patients required ICU admission and one in five required mechanical ventilation [5]. Older age, history of ventricular tachycardia, lower platelet count, use of antiplatelet drugs - P2Y12 inhibitors, lower albumin and higher troponin T levels were identified as independent predictors of in-hospital mortality [5].

Genetic differences may contribute to individual variations in the immune response to the virus [13]. This has been shown by a Genomewide Association Study (GWAS) involving 1610 COVID-19 patients from seven hospitals in Italy and Spain and 2205 control participants [14]. A novel susceptibility locus was detected at a chromosome 3p21.31 gene cluster, and a relationship was found between the ABO blood-group system and the COVID-19 infection. Patients with blood group A had higher risk of more severe illness than other blood groups [odds ratio (OR) 1.45, 95% confidence interval (CI) 1.20–1.75] whereas blood group O was associated with a protective effect (OR 0.65, 95% CI 0.53–0.79). Furthermore, a severe COVID-19 phenotype has been observed amongst the BAME (Black and Minority Ethnic) population, where the chronic systemic inflammation induced by preexisting metabolic syndrome is believed to be significantly enhanced by COVID-19 infection [15].

## HYPERTENSION AND ANTIHYPERTENSIVE TREATMENT IN CORONAVIRUS DISEASE 2019

As mentioned above, early after the outbreak of the pandemic, several studies indicated hypertension as a predictor of adverse outcome in COVID-19 patients. Some later studies that made use of adjusted models questioned this conclusion and regarded only age, diabetes, chronic obstructive pulmonary disease and chronic kidney disease as independent predictors of the severity of the COVID-19 infection [10]. However, the association between hypertension and COVID-19 severity is now well recognized. Richardson *et al.* studied the clinical characteristics of 5700 hospitalized COVID-19 patients (mean age 63 years, 39.7% women) from New York area in the United States [11]. Hypertension was the most frequent concomitant comorbidity (prevalence of 56.6%) followed by obesity (41.7%) and diabetes (33.8%). Overall, 21% patient died, 14.2% required intensive care, 12.2% were subjected to invasive ventilation and 3.2% to renal replacement therapy. Similar data have been reported in other studies, and an independent association between hypertension and risk of COVID-19 (OR: 2.29, *P* < 0.001) has recently been confirmed in a meta-analysis of six studies that included 1558 patients [16].

COVID-19 causes severe ARDS by binding and entering target epithelial lung and other cells through the peptide angiotensin-converting enzyme (ACE)-2. As treatment with the renin--angiotensin system (RAS) blockers has been shown to upregulate ACE-2 at the cell surface [17], shortly after the outbreak of COVID-19, there were concerns that use of ACE inhibitors or angiotensin receptor blockers to treat hypertension, heart failure, chronic kidney disease or the postmyocardial infarction state might favour the risk or the severity of the COVID-19 infection. Two large-scale studies, however, have shown that chronic treatment with either ACE inhibitors or angiotensin receptor blockers do not significantly modify the risk or the severity of COVID-19 infection after adjustment for potentially confounding variables [12,18], which disposes also of the hypothesis that as ACE-2 metabolizes angiotensin II into a vasodilator substance, these drugs might exert a protective effect [17]. This view is also supported by a recent study suggesting that ACE inhibitors and ARBs are not associated with an increased risk of organ failure in COVID-19 [19], as well as by a recent meta-analysis of a large number of studies [20], which reassured on the safety of the large use of these drugs to protect patients in a number of important diseases. This is clinically crucial as in these diseases, RAS blockers has a life-saving role and their discontinuation is accompanied by a marked increase of cardiovascular outcomes and mortality [12,17,18,20]. The protective effect of these drugs may include their reported anti-inflammatory influence as an excessive inflammatory reaction and a pronounced increase of a number of inflammatory markers have been shown to adversely affect the course of the disease and prognosis [21].

Evidence that previous chronic exposure to RAS blockers does not modify the risk and severity of COVID-19 leaves some important questions unanswered. One of them is whether antihypertensive treatment in general, and RAS blocker administration in particular, favourably affects the course of COVID-19. Although some observational studies speak in favour of this possibility [12,18,22], this question can only find a conclusive answer in the context of randomized trials that avoid the limitations associated with the baseline variable imbalance and confounding by indication typical of observational studies [23,24]. One such trial is so far available with no evidence that administration of RAS blockers at the beginning of COVID-19 infection modifies the course of the disease compared to placebo [22].

## CLINICAL COURSE OF CORONAVIRUS DISEASE 2019

**FIGURE 1:**
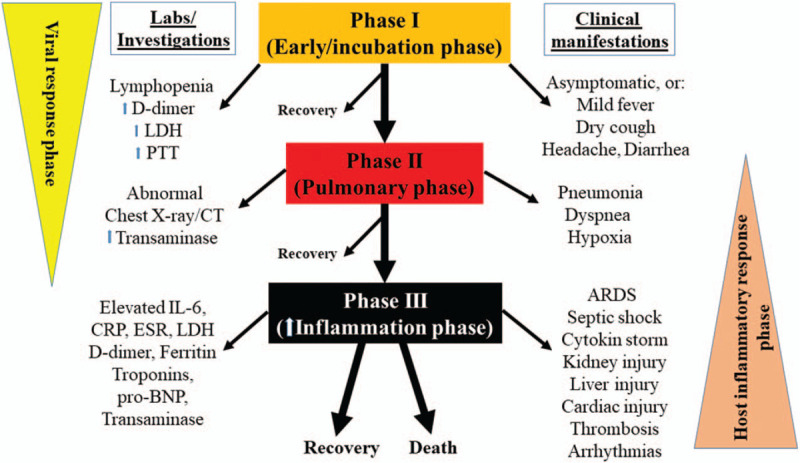
Clinical course with various phases of coronavirus disease 2019. ARDS, acute respiratory distress syndrome; CRP, C-reactive protein; CT, computed tomography; ESR, erythrocyte sedimentation rate; IL-6, interleukin 6; LDH, lactate dehydrogenase; pro-BNP, pro-brain natriuretic peptide.

After exposure to SARS-CoV-2 some patients may develop infection and some not. Among those infected, the clinical manifestations vary, and the disease course can generally be divided into several phases (Fig. 1). The majority of symptomatic COVID-19 patients have mild self-terminating disease courses characterized by fever, dry cough, shortness of breath, hemoptysis, muscle and/or joint pain, headache, dizziness, diarrhea and nausea [25–27]. However, in more severe cases, the coronavirus causes pneumonia, confirmed by a chest X-ray or chest CT (computed tomography), which may lead to ARDS. The severe forms of the diseases also include a variable involvement of organs other than the lungs with in some cases multiorgan failure. Lung inflammation and subsequent respiratory failure because of COVID-19-associated pneumonia is a major cause of in-hospital deaths [28,29] but other organs, especially the heart, the microvasculature and the kidney importantly participate in the severe clinical pictures, which makes COVID-19 a systemic disease. The factors and mechanisms that favour or lead progression of COVID-19 from milder to severe stages is believed to be largely dependent on the effectiveness of the early immune response. In detail, coronavirus initially binds to ACE-2 receptor on alveolar macrophages and epithelial cells with viral replication in airway epithelial cell. The adaptive response during incubation and nonsevere phases tries to eliminate the virus. However, when the initial protective immune response fails, the disease progresses to more severe forms with organ involvement. In the lungs, the pneumocyte damage, hypoxia and capillary leak occur [30,31], the innate inflammation being largely mediated by pro-inflammatory macrophages and granulocytes. This phase can further progress to a cytokine storm [cytokine release syndrome (CRS)] because of the host-inflammatory response, leading to worsening of respiratory symptoms, multiorgan failure and hemodynamic instability. The cytokines involved in COVID-19 are, among others, interleukins (IL-6, IL-1β, and tumor necrosis factor α), which are believed to be responsible for septic shock and multiorgan failure, including myocardial damage and circulatory failure [30,31]. An important role in the overall clinical picture of a severe COVID-19 infection is believed to be played by virus-related endothelial dysfunction and damage, the consequence of which can be systemic intravascular coagulation and embolism (Fig. 1).

## CARDIOVASCULAR COMPLICATIONS OF CORONAVIRUS DISEASE 2019

COVID-19 may cause acute cardiovascular organ damage, and lead to postrecovery sequelae [1–5,32]. Cardiovascular involvement during the infection may be because of the virus toxicity or to a dysregulation of the inflammatory or immunological responses leading to a cytokine storm. However, the likelihood in the severe or systemic COVID-19 phases, multiorgan involvement may appear and progress independently of a cytokine storm. There is evidence that preexisting organ damage is associated with a more severe disease course of COVID-19, as recently exemplified by the association that has been found between the extent of coronary artery calcification and a worse prognosis of hospitalized COVID-19 patients [33]. It has been speculated that in the COVID-19 infection, the magnitude of the immune response, the degree of endothelial dysfunction and myocardial stress can be exacerbated by a preexisting subclinical coronary atherosclerosis [33].

## ACUTE CARDIOVASCULAR SYNDROME IN CORONAVIRUS DISEASE 19

**FIGURE 2:**
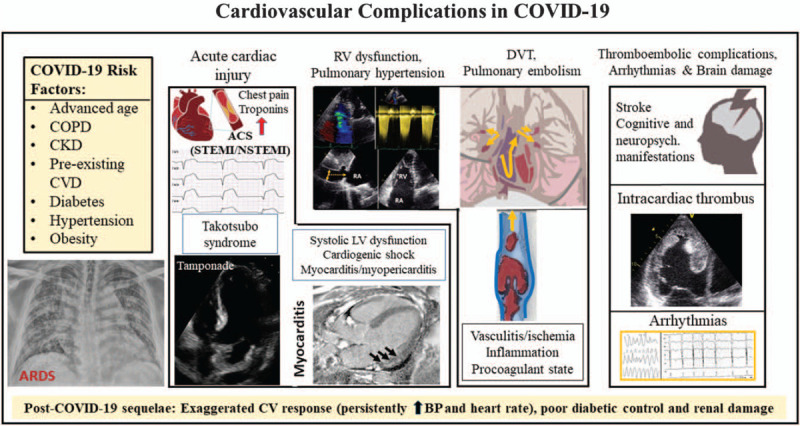
Overview of acute cardiovascular complications in coronavirus disease 2019. ACS, acute coronary syndrome; CVD, cardiovascular disease; CKD, chronic kidney disease; COPD, chronic obstructive pulmonary disease; DVT, deep venous thrombosis.

The cardiac involvement of COVID-19 is variable and ranges from mildly elevated cardiac biomarkers to acute cardiogenic shock and sudden cardiac death. A typically acute cardiac syndrome (COVID cardiomyopathy) [1–5,32,34] includes acute cardiac injury, thromboembolic complications, atrial and ventricular arrhythmias, hemodynamic instability and sudden cardiac death **(**Fig. 2) [2,3,32]. In a recent study of 102 confirmed COVID-19 patients, Xu *et al.*[34] found different patterns of cardiac involvement, which included elevated myocardial enzyme levels in 53.9%, cardiac dysfunction in 41.2%, tachycardia in 19.6% patients and acute cardiac injury in 9% patients.

**FIGURE 3:**
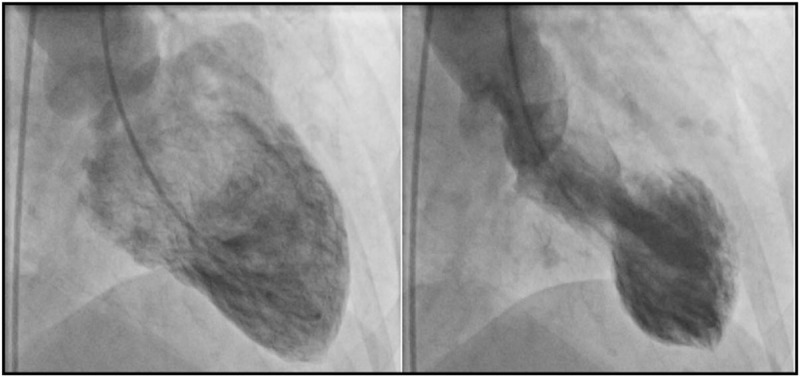
Ventriculography images of a coronavirus disease 2019 patient with Takotsubo cardiomyopathy. End-diastolic frame (left) and end-systolic frame (right) showing typical obstruction of the basal part of the heart with apical ballooning.

**FIGURE 4:**
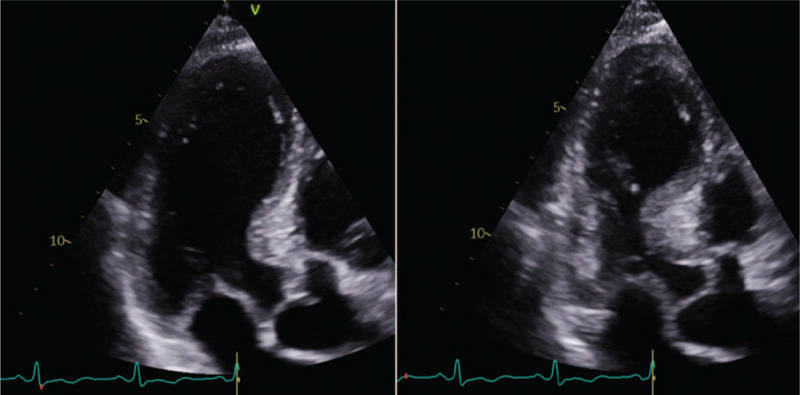
Transthoracic echocardiography images of a coronavirus disease 2019 patient with Takotsubo cardiomyopathy. Apical long-axis views. End-diastolic frame (left) and end-systolic frame (right) showing typical obstruction of the basal part of the heart and apical ballooning.

**FIGURE 5:**
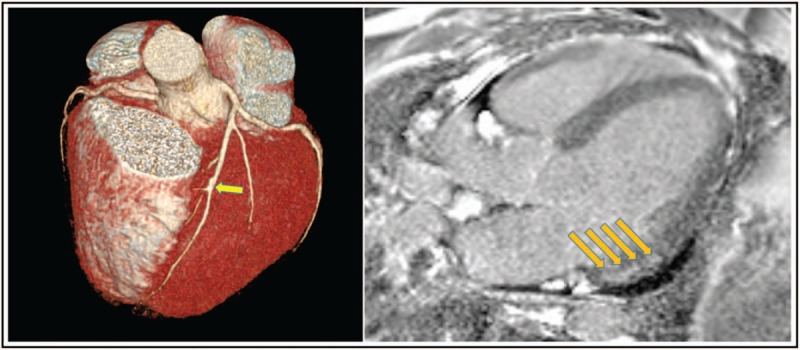
Cardiac computed tomography (left) and magnetic resonance (right) images of a coronavirus disease 2019 patient with chest pain and elevated cardiac troponins (1088-54 ng/l; normal range <15 ng/l) and left ventricular ejection fraction 44%. Cardiac CT shows open coronary artery, only a minor calcified lesion in mid-LAD (arrow). Cardiac MR shows late gadolinium enhancement (arrows) in the left ventricular inferolateral wall, mid-myocarrdium/subepicardial, suggesting subacute perimyocarditis. CT, computed tomography; LAD, left anterior descending artery; MR, magnetic resonance.

The clinical presentation of acute cardiac injury may be chest pain, shortness of breath, syncope/near syncope, tachycardia, elevated myocardial troponins and natriuretic peptides, regional wall motion abnormalities or global left ventricular (LV) dysfunction on echocardiography and ST segment depression or elevation or T-wave abnormalities on ECG. Cardiac injury in COVID-19 patients may occur both with and without acute obstruction of the coronary arteries (irrespective of a preceding atherosclerotic coronary artery disease), which portends poor prognosis [35]. It is believed that virally induced thrombi may result in acute coronary syndromes (NSTEMI or STEMI) [36] although some other causes have been described: type 2 myocardial infarction because of myocardial demand--supply mismatch secondary to tachycardia, hypotension and hypoxemia; microvascular damage because of diffuse microembolism; cardiotoxic effects of an inflammation-induced systemic cytokine storm, possibly by a cytokine pattern different from that made by ’classical’ cytokines, and stress-induced cardiomyopathy (also termed Takotsubo syndrome) **(**Figs. 3 and 4) [2,36]. Acute heart failure syndromes [systolic right ventricular (RV) and LV dysfunction with or without cardiogenic shock], myopericarditis and myocarditis **(**Fig. 5**)** are also common and may be induced by invasion of the myocardium by the coronavirus (primary cardiomyocyte injury) or by an enhanced inflammatory response, and thus a cytokine-mediated injury. Recently, severe cases of acute perimyocarditis with subsequent cardiac tamponade in patients with COVID-19 infection have also been reported [37,38]. An early and focused cardiac ultrasound allowed the diagnosis of cardiac tamponade to be timely made, prompting a lifesaving acute pericardial drainage [38]. The European and American Societies of Cardiology recommend a focused cardiac ultrasound study (FoCUS) of the COVID-19 patients and consider bedside critical care echocardiography as effective options to screen for cardiovascular complications in the COVID-19 infection [8,39].

COVID-19 may also affect the RV, but compared with LV, RV has been less extensively studied. The tricuspid annular plane systolic excursion (TAPSE) is often preserved in hospitalized COVID-19 patients [40]. Furthermore, in a cohort of COVID-19 ARDS patients studied in a UK tertiary hospital, a specific phenotype of RV radial dysfunction with sparing of longitudinal function was observed [41]. Notably, in these patients, the RV--pulmonary artery coupling, as reflected by the fractional area change divided by RV systolic pressure (RV afterload), was an important marker of disease severity. RV longitudinal strain has also been identified as a powerful predictor of mortality in COVID-19 patients [42].

Acute cardiac injury in COVID-19 is associated with poor prognosis. Shi *et al.*[35] showed that, compared with patients without cardiac injury (*n* = 334, 80.3%), patients with acute cardiac injury (*n* = 82, 19.7%) had a significantly higher risk of in-hospital mortality from the time of symptom onset (hazard ratio 4.26, 95% CI 1.92–9.49) or hospital admission (hazard ratio 3.41, 95% CI 1.62–7.16).

Limited data are available on the prevalence of spontaneous coronary dissection in COVID-19 patients. The acute cardiac injury, which becomes manifest as Takotsubo syndrome in COVID-19 female patients should be differentiated from spontaneous coronary dissection, which is linked to a systemic inflammatory response, hemodynamic stress and hypertension [43], risk factors that are highly prevalent in COVID-19. There is also lack of evidence on the COVID-related native and prosthetic valve endocarditis. It is important to highlight that COVID-19 can cause decompensation of an underlying heart failure. This may lead to a mixed shock and require specific therapeutic approaches. Differentiating COVID-19-related acute lung injury from patients with acute pulmonary edema and preexisting heart failure is also crucial as continuous positive airway pressure (CPAP) is contraindicated in the first group of patients and beneficial in the second group of patients. Finally, it is also important to note that the clinical presentation of some fulminant myocarditis, such as giant cell myocarditis may resemble COVID myocarditis. An endomyocardial biopsy should be considered in patients presenting with higher burden of life-threatening arrhythmias and cardiogenic shock and a negative test for COVID-19 infection. However, the two conditions may also coexist.

## THROMBOEMBOLIC COMPLICATIONS

COVID-19 is a hyper-coagulant state in which platelet activation, neutrophil recruitment, disseminated intravascular coagulation and fibrinogen degradation are common. In addition, localized inflammation because of direct viral invasion of pericytes and vascular endothelium may induce microvascular dysfunction. All these factors lead to thromboembolic complications, such as arterial thromboembolism, intracardiac thrombosis, microvascular thrombi, pulmonary embolism, cerebral venous thrombosis and stroke (Fig. 2) [4,44–46]. Intracardiac thrombosis following prolonged treatment with extracorporeal membrane oxygenation has also been reported [47].

## ARRHYTHMIAS

In COVID-19 patients, arrhythmias may occur because of cardiac injury (inflammation, ischemia, stress); the detrimental systemic effects of COVID-19 (dehydration, hypoxia, electrolyte abnormalities) or the pro-arrhythmic effects of the drugs given to the patients before or during the infection [48–50]. Approximately 6–17% of COVID-19 patients develop arrhythmias, and this percentage increases markedly (up to 44%) in patients treated in ICU [51]. Sinus tachycardia is common in COVID-19 patients, in whom it becomes most frequently manifest as palpitations, whereas the most frequent pathological arrhythmias are atrial fibrillation, atrial flutter and ventricular arrhythmias [25,51]. In hospitalized patients with COVID-19, the prevalence of atrial fibrillation was reported to range between 9 and 21% [52,53]. Bradyarrhythmias were also not rare, that is, there was 8% of patients with sinus bradycardia and another 8% with complete heart block [53]. In a study of COVID-19 patients from Spain, the prevalence of previously known atrial fibrillation was 18.8% whereas new-onset atrial fibrillation involved 7.5% of the patients. New-onset of atrial fibrillation during hospital was an independent predictor of in-hospital embolic events [50].

## ROLE OF BIOMARKERS IN CORONAVIRUS DISEASE-2019 WITH AN ACUTE CARDIOVASCULAR SYNDROME

Common biomarkers that are elevated in COVID-19 are cardiomyocyte-specific biomarkers [high-sensitivity troponin and N-terminal pro-brain natriuretic peptide [NT-pro-BNP]] and coagulation (fibrinogen, D-dimer, prothrombin time, partial thromboplastin time) and inflammatory markers (C-reactive protein, interleukin-6, lactate dehydrogenase and ferritin) (Fig. 1) [3]. Lymphopenia is also common. Procalcitonin levels are usually low, which helps to differentiate COVID-19 from acute bacterial infections [1]. Among the aforementioned biomarkers, the elevated cardiac troponins and pro-BNP are associated with acute cardiac injury and worse prognosis [25,35–38,43–45,47,51,54]. It is common opinion that worse prognosis may also be associated with elevated serum levels of pro-inflammatory chemokines and cytokines (interleukins, interferon-gamma, interferon-inducible protein and monocyte chemoattractant protein-1), which recruit monocytes, macrophages and T cells at the site of infection, thereby favouring tissue damage and destruction. The complexity of the processes favouring and opposing the virus is further emphasized by the evidence that severe ARDS in COVID-19 patients may also be associated with cellular immune deficiency [25,34].

Finally, a recent multicenter study has found the presence of myocarditis (multiple foci of inflammation) in cardiac tissue from autopsies in COVID-19 patients. An increased interstitial macrophage infiltration was present in majority of the cases, while multifocal lymphocytic myocarditis was found only in few cases [55].

## BLOOD PRESSURE AND HEART RATE SEQUELAE AFTER RECOVERY FROM CORONAVIRUS DISEASE 2019

**FIGURE 6:**
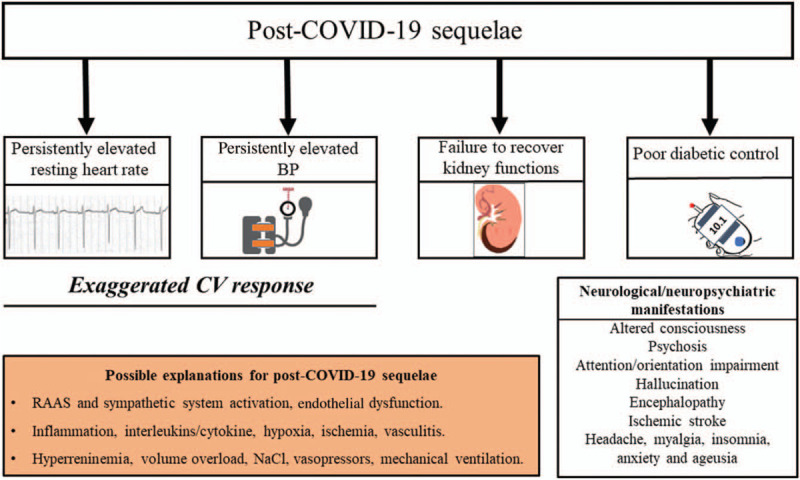
Post- coronavirus disease 2019 sequelae: persistently elevated blood pressure and resting heart rate, kidney injury, poor glycemic control and neurological manifestations, and their possible explanations suggested in the literature. BP, blood pressure; RAAS, renin--angiotensin--aldosterone system.

COVID-19 survivors may sometimes show persistently elevated blood pressure (BP) and high resting heart rate (sinus tachycardia) in the convalescent period **(**Fig. 6**)**, which adds to the concern raised by the evidence that COVID-19 may lead to long-lasting or semi-permanent post-COVID alterations of the heart, the lungs and other organs [56].

The cause and clinical significance of the above-mentioned BP and heart rate abnormalities after recovery from COVID-19 are yet to be explored, although some hypotheses can already be made [57, Wasim *et al.*, 2020; unpublished observations]. For example, a persistent elevation of BP and heart rate after an apparent recovery from COVID-19 might originate from an activation of the RAAS or the sympathetic nervous system, possibly favoured by damage and dysfunction of reflexes restraining sympathetic and enhancing parasympathetic tone via virus-related neural damage. However, prolonged periods of mechanical ventilation and associated sedation, repeated administration of inotropic agents, fever, hypoxia, inflammation (pro-inflammatory cytokines/interleukins), ischemia, vasculitis may all be additional contributing factors [57, Wasim *et al.*, 2020; unpublished observations]. In addition, preliminary results based upon case series have recently indicated that patients with COVID-19 under prolonged intensive care tend to develop hyperreninemia combined with hypernatremia, hyperchloremia and volume overload [57]. After recovery from the COVID-19 infection, these changes may lead to persistently elevated BP in previously normotensive individuals or favour poor BP control in patients with a preexisting controlled hypertensive state.

## DIABETES AND CORONAVIRUS DISEASE 2019 INFECTION

Diabetes and COVID-19 may have a bidirectional relationship in the same manner as hypertension; that is, diabetes is associated with an increased risk of COVID-19 severity and adverse outcome, and COVID-19 can worsen glycemic control and/or cause severe hyperglycemic crisis and metabolic complications in hospitalized patients with previously known or newly detected diabetes [58,59]. Recent case series have also indicated that COVID-19 can trigger diabetic ketoacidosis among patients with previously known diabetes, as well as new-onset diabetes can be the first clinical presentation of COVID-19 patients [59]. Indeed, previous experiences from SARS coronavirus suggested that acute damage to the β-cells of the pancreas might lead to insulin deficiency and result in diabetic ketoacidosis in patients with known diabetes, or simply trigger diabetes [60]. Similar to the association between COVID-19 and hypertension, increased inflammation-induced cytokine/interleukins may also be important for the association between COVID-19 and diabetes.

Sardu *et al.*[61] recently showed that hyperglycemia in hospitalized COVID-19 patients treated with insulin infusion had a lower risk of severe illness than those who were not treated with insulin infusion. They postulated that insulin infusion might be an effective measure for glycemic control in COVID-19 patients.

## ACUTE KIDNEY INJURY AND CORONAVIRUS DISEASE 2019

Acute kidney injury is common in hospitalized patients with COVID-19 and associated with higher mortality [62]. Direct invasion of the kidney tissue by the coronavirus 2 with subsequent tubular injury as well as endothelial damage may contribute to acute kidney injury [62,63]. Hematuria, proteinuria and leukocyturia are common urine abnormalities. Of note, these abnormalities may occur even in the presence of normal serum creatinine, which indicates that there may be other mechanisms in addition to acute tubular injury. In a large retrospective study of 3993 patients hospitalized with COVID-19 infection, acute kidney injury occurred in 46% patients. Among these, 19% (*n* = 347) required dialysis [62]. Acute kidney injury was particularly common (76%) in those patients who were admitted to intensive care (*n* = 976; 24%). The risk of in-hospital mortality was nearly six-fold higher in patients with acute kidney injury compared with those without acute kidney injury. Furthermore, COVID-19 patients with underlying diabetes and hypertension may be more prone to develop acute kidney injury. In the study of Chan *et al.*[62], kidney function did not recover to baseline in nearly one-third of survivors by hospital discharge. The most striking findings were that among patients with acute kidney injury, only 30% survived who had their kidney functions recovered. However, in COVID-19 survivors, the exact mechanism of acute kidney injury, as well as chronic/persistent renal impairment is not well understood. Future studies may provide useful insights on this topic and enable potential therapeutic interventions.

## CORONAVIRUS DISEASE 2019 AND COGNITIVE AND NEUROPSYCHIATRIC MANIFESTATIONS

Evidence is available that COVID-19 patients may exhibit, a wide range of neurological/cognitive and neuropsychiatric manifestations [55]. The mechanisms of the involvement of cerebrovascular system in the coronavirus infection are still under investigation but factors, such as direct brain invasion by the coronavirus, virus-related damage of peripheral motor and autonomic nerves (suggested by symptoms, such as disgeusia and anosmia as well as by cases of Guillain--Barrè syndrome) a hyperinflammatory response, cytokine storm, cerebral ischemia secondary to endothelial dysfunction, vasculitis and coagulopathy, and prolonged use of invasive ventilation, and associated sedation have been offered as mechanistic background, and are thus all potential candidates [64,65]. Neurological symptoms may have a rather specific nature or be mild and nonspecific, such as headache, myalgia, insomnia and anxiety. They can also be more severe and lead to altered consciousness, posttraumatic stress syndrome, neuroinflammatory syndromes, psychosis, attention and orientation impairment, visual or auditory hallucinations, encephalopathies, ischemic strokes or Guillain--Barrè syndrome [64–74]. It is important to note that elderly people with chronic neurogenerative disease/dementia are more frequently presented with atypical symptoms, such as altered mental status, confusion, agitation, disorientation and loss of appetite [75–77]. Also, in the COVID-infected people, the severity of neurological manifestations tends to be related to the severity of underlying cardiovascular risk factors in most individual patients. To date, the long-term neurological sequelae in COVID-19 survivors are poorly known and they will have to be addressed more in depth by future studies, possibly via a larger use of a neuroimaging approach.

## FUTURE STUDIES

Currently, a few prospective studies have been conducted on the extent, progression and recovery of organ damage in COVID-19 patients; the available evidence being mainly based on single case-reports or case series, or smaller retrospective observational studies with short follow-up. There is need for larger multimodality imaging studies. Contrast-enhanced echocardiography as a bedside procedure may have valuable clinical value in diagnosing and managing suspected cardiac masses in hospitalized patients with COVID-19, who have higher incidence of venous thromboembolism. Furthermore, in the follow-up of patients recovered from COVID-19, contrast-enhanced echocardiography and strain imaging may be useful for assessment of myocardial perfusion, coronary flow reserve and subclinical myocardial dysfunction. This is particularly relevant for patients who have preserved LV ejection fraction. CMR is also an important imaging modality for assessing myocardial inflammation, scar/thinning, late gadolinium enhancement during acute hospitalization as well as after recovery from COVID-19. In a CMR study of 100 patients who recently recovered from COVID-19 infection, Puntmann *et al.*[78] showed that vast majority of patients (78%) had various degree of cardiac involvement and abnormal CMR finding including raised myocardial native T1 and T2, and fibrosis as reflected by pericardial and myocardial and late gadolinium enhancement. Of note, 60% (*n* = 60) had an ongoing myocardial inflammation, although CMR was performed in average 71 days after COVID-19 diagnosis. The presence of myocardial inflammation was independent of previous comorbidities, disease severity and time interval between COVID-19 diagnosis and CMR.

RV dysfunction and elevated systolic pulmonary artery pressure can also occur in COVID-19 patients and are associated with poor outcome [42,79]. Acute respiratory failure/ARDS, inflammation, ischemia and vasculitis may be reasons for new-onset pulmonary hypertension in COVID-19 patients. Although case reports have indicated that elevated systolic pulmonary artery pressure and RV dysfunction may be reversible [Wasim *et al*., 2020; unpublished observations], their long-term prognostic implications remain currently unknown.

Regarding hypertension and antihypertensive treatment, there is need for on-treatment more powerful trials on ACE inhibitors versus ARBs, and BP control versus no control, as well as post-COVID sequelae. Most importantly, available data in the literature is derived from hospitalized patients who were generally older and had high cardiovascular disease burden. Nonhospitalized COVID-19 patients who are younger, have lower cardiovascular disease burden and recovered at home, have not been studied sufficiently, and should be the target of future research with regard to long-term cardiovascular complications.

Finally, during the COVID-19 pandemic, there have also been growing concerns that patients with cardiovascular diseases may not seek healthcare system timely because of fear of contracting COVID-19 infection. This may contribute to significant increase in cardiovascular morbidity and mortality in post-COVID-19-era worldwide, which we need to be prepared for.

## CONCLUSION

In COVID-19 patients, the cardiovascular system is profoundly affected by direct effect of coronavirus as well as by the subsequent dysfunctional inflammatory response. Patients may develop acute cardiovascular complications, such as acute COVID-cardiomyopathy, cardiac injury, thromboembolic complications and/or arrhythmias, which are all associated with higher mortality. COVID-19 survivors may also show persistently elevated BP and sinus tachycardia. This clinical entity has a complex and multifactorial cause involving dysregulation of inflammatory processes with systemic cytokine storm, immunological alteration, and hyperreninemia, volume overload and vasopressor treatment in ICU setting. In case of a persistently elevated BP and sinus tachycardia, a 24-h ambulatory BP monitoring should be performed to evaluate the true hypertension state, BP control and assess nondipping BP pattern and nocturnal heart rate response. On the basis of the available evidence in the literature, it seems that COVID-19 leads to worsening of hypertension, diabetes and renal damage. Close follow-up and careful optimization of antihypertensive treatment is essential to avoid long-term cardiovascular complications. Finally, cerebrovascular sequelae, such as persistent cognitive and neuropsychiatric alterations are also frequently observed in COVID-19 patients. These require careful clinical assessment, treatment and close follow-up.

## ACKNOWLEDGEMENTS

S.S. and G.M. wrote the first draft of the manuscript. M.T., T.H.L. and G.G. revised the manuscript. All authors approved the final submission.

### Conflicts of interest

There are no conflicts of interest.
